# Landscape of sialylation patterns identify biomarkers for diagnosis and prediction of response to anti-TNF therapy in crohn’s disease

**DOI:** 10.3389/fgene.2022.1065297

**Published:** 2022-11-14

**Authors:** Chenglin Ye, Sizhe Zhu, Yuan Gao, Yabing Huang

**Affiliations:** ^1^ Department of Pathology, Renmin Hospital of Wuhan University, Wuhan, China; ^2^ Department of Otolaryngology-Head and Neck Surgery, Tongji Hospital, Tongji Medical College, Huazhong University of Science and Technology, Wuhan, China

**Keywords:** crohn’s disease, anti-TNF therapy, sialylation, immune infiltration, bioinformatics analysis

## Abstract

Crohn’s disease (CD), a subtype of inflammatory bowel disease (IBD), causes chronic gastrointestinal tract inflammation. Thirty percent of patients do not respond to anti-tumor necrosis factor (TNF) therapy. Sialylation is involved in the pathogenesis of IBD. We aimed to identify potential biomarkers for diagnosing CD and predicting anti-TNF medication outcomes in CD. Three potential biomarkers (SERPINB2, TFPI2, and SLC9B2) were screened using bioinformatics analysis and machine learning based on sialylation-related genes. Moreover, the combined model of SERPINB2, TFPI2, and SLC9B2 showed excellent diagnostic value in both the training and validation cohorts. Importantly, a Sial-score was constructed based on the expression of SERPINB2, TFPI2, and SLC9B2. The Sial-low group showed a lower level of immune infiltration than the Sial-high group. Anti-TNF therapy was effective for 94.4% of patients in the Sial-low group but only 15.8% in the Sial-high group. The Sial-score had an outstanding ability to predict and distinguish between responders and non-responders. Our comprehensive analysis indicates that SERPINB2, TFPI2, and SLC9B2 play essential roles in pathogenesis and anti-TNF therapy resistance in CD. Furthermore, it may provide novel concepts for customizing treatment for individual patients with CD.

## Introduction

Crohn’s disease (CD) is a significant type of inflammatory bowel disease (IBD) that is characterized by a chronic inflammatory condition that can affect any area of the gastrointestinal tract (Adegbola et al., 2018; Cushing and Higgins, 2021). Approximately 5% of people worldwide are affected by CD (Kaplan, 2015). In China, the estimated incidence rate is 0.51–1.09 per 100,000 people (Ng et al., 2017; Li et al., 2019a). Currently, there are no curative treatments for CD. The tumor necrosis factor (TNF) inhibitor infliximab, the first biological response modifier, was licensed for treating CD in 1998 and increased patient response and remission rates (Adegbola et al., 2018). Up to 30% of patients do not respond to anti-TNF medications, and 50% of patients who initially benefit from these medicines lose clinical improvement within the first year, necessitating dosage increase or therapy change (Adegbola et al., 2018; Ye et al., 2022). Therefore, exploring effective therapeutic strategies for patients with CD is crucial.

Sialylation involves the addition of sialic acid to the terminal end of glycoproteins, a biologically significant alteration involved in microbial dysbiosis, gut inflammation, and immunological responses (Li and Ding, 2019; Giron et al., 2020). A recent study reported that sialylation of intestinal mucus by ST6GALNAC1 is essential for commensalism and bacterial metabolite balance as well as intestinal barrier integrity in IBD. The integrity of the mucus is preserved by ST6GALNAC1-mediated sialylation, which protected MUC2 from being degraded by certain bacterial-secreted mucinases (Yao et al., 2022). Meanwhile, a local release of free sialic acid during inflammation is probably facilitated by the increase in sialylation of intestinal mucins during colitis. This leads to an overgrowth of *E. coli*, which exacerbates the pro-inflammatory response by intestinal dendritic cells (Parker et al., 1995; Huang et al., 2015). However, a comprehensive analysis of multiple sialylation-related gene and their roles in CD is lacking. Therefore, exploring expression patterns and functions of sialylation-related gene may help to understand the heterogeneity and pathogenesis of CD.

This study comprehensively analyzed the expression patterns and functions of sialyation-related genes in CD using bioinformatics and machine learning. First, patients with CD were classified into two subtypes based on the expression of differentially expressed sialylation-related genes. The immune infiltration level and anti-TNF therapy response of patients with CD of the two subtypes were analyzed. Weighted gene co-expression network analysis (WGCNA), least absolute shrinkage and selection operator (LASSO) regression, random forest (RF), and support vector machine recursive feature elimination (SVM-RFE) were applied to further screen biomarkers for anti-TNF therapy response. Moreover, a scoring system, the Sial-score, was established to predict the response to anti-TNF therapy in patients with CD before or after treatment.

## Method

### Data collection

Using the search terms “Crohn’s Disease and anti-TNF” or “infliximab”, gene expression cohorts for CD were retrieved from the Gene Expression Omnibus database. The following cohorts were obtained: GSE16879 (213 CD inflamed tissues before and after infliximab treatment and 13 normal tissues) (Arijs et al., 2009), GSE111761 (lamina propria mononuclear cells isolated from six CD tissues) (Schmitt et al., 2019), GSE42296 (peripheral blood samples were obtained from 20 patients with CD) (Mesko et al., 2013), GSE107865 (whole blood samples were collected from 22 patients with CD) (Gaujoux et al., 2019), GSE102133 (55 inflamed CD tissues and 12 normal tissues) (Verstockt et al., 2019), and GSE179285 (47 inflamed CD tissues and 31 normal tissues) (Keir et al., 2021). The GSE16879, GSE111761, GSE42296, and GSE107865 cohorts contained clinical information on whether patients responded to infliximab treatment. According to the clinician’s assessment, the patients were classified according to their response to infliximab based on endoscopic and histologic findings 6 or 14 weeks after the first infliximab treatment. Sialyation-related genes were obtained from the GeneCards database using the search term “sialyation”. Genes with relevance scores >1 were selected for further analysis.

### Differential analysis and unsupervised clustering

The Limma R package was used for differential analysis (Ritchie et al., 2015). Differentially expressed genes (DEGs) were classified as genes with an adjusted *p*-value <0.05 and | log2 (fold-change)| > 1.0. Sangerbox3.0 (http://vip.sangerbox.com/) was used to perform a consensus clustering algorithm using the R package ConsensusClusterPlus (Wilkerson and Hayes, 2010; Shen et al., 2022) to identify distinct subtypes. This was repeated 1,000 times to confirm clustering stability. Supplementary Table S1 shows the group information following the unsupervised clustering of cohorts.

### WGCNA and single-sample gene set enrichment analysis

WGCNA was used to identify the related modules. The minimum number of module genes was set to 30, the parameter deepslip was set to 4, and the mergeCutHeight was set to 0.25. The hierarchical clustering dendrogram summarizes the gene modules with different colors (Langfelder and Horvath, 2008; Ye et al., 2021). Based on the cutoff criteria, genes with high connections in clinically relevant modules were identified as hub genes. The ClueGO plug-in was used to analyze biological functions in Cytoscape 3.8.2.

The relative level of immune cell infiltration was estimated using single-sample gene set enrichment analysis (ssGSEA). The gene signatures of the immune cells are listed in Supplementary Table S2 (Bindea et al., 2013).

### Screening biomarkers based on machine learning

LASSO regression was applied to select potential biomarkers using the glmnet R package (Friedman et al., 2010). Binomial distribution variables were then used in the LASSO classification coupled with one standard error lambda value for the minimum criterion. RF, a tree-based ensemble of tree-structured classifiers, was created using the package “randomForest” using least error regression trees for clinical feature genes. The importance of the factors was ranked using “Mean Decrease Accuracy” and “Mean Decrease Gini”. SVM is a type of generalized linear classifier that uses supervised learning to categorize binary data (Huang et al., 2018). SVM-RFE, a SVM-based algorithm, was applied to select relevant genes through nonlinear kernels (Sanz et al., 2018).

### Construction of the predicting score system

The expression of potential biomarkers was used to develop a scoring system based on principal component analysis (PCA) to predict the response to anti-TNF medication. The Sial-score was calculated as follows: Sial-score = ∑PC1i, where i is the potential biomarker.

## Results

### Identification of sialyation-related DEGs

**FIGURE 1:**
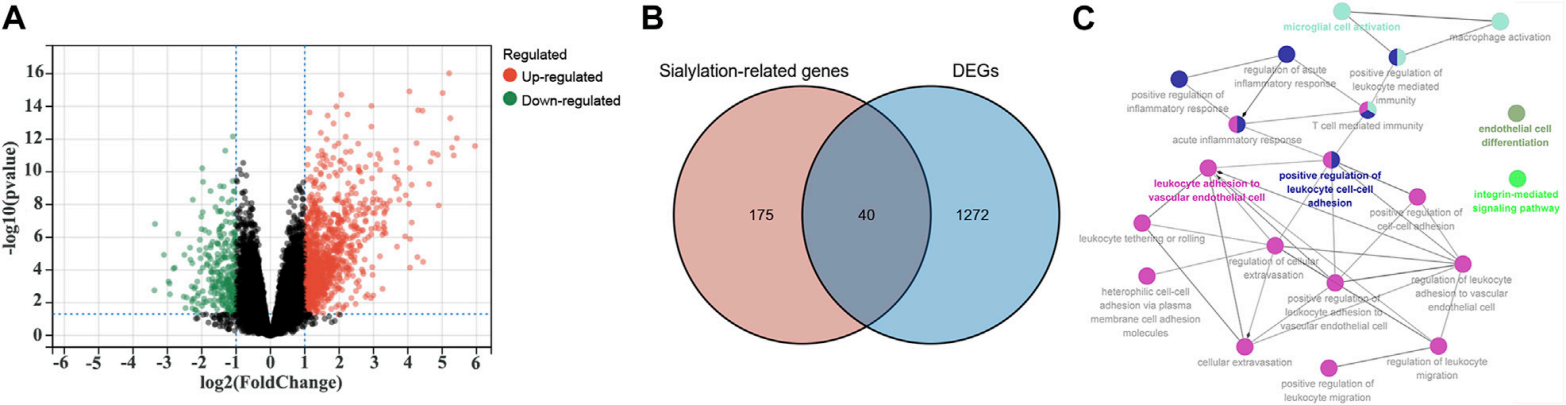
Identification of Sialylation-related DEGs in GSE16879 cohort. **(A)** A volcano plot of DEGs between CD and normal samples. **(B)** Intersection of DEGs and Sialylation-related genes. **(C)** Biological functions of Sialylation-related DEGs. Each color represents a different functional group. Each node represents a GO term and each line represents a correlation between different terms.

Differential expression analyses were performed using the limma R package in the GSE16879 cohort to identify sialyation-related DEGs between the CD and normal samples. A volcano plot of the DEGs is presented in Figure 1A. 215 sialyation-related genes were used to overlapped with DEGs, a total of 40 sialyation-related DEGs were obtained, including 38 upregulated and two downregulated (Figure 1B). Furthermore, ClueGO was used to explore the biological functions of the sialyation-related DEGs. As shown in Figure 1C, sialyation-related DEGs were significantly enriched in leukocyte adhesion to vascular endothelial cells, positive regulation of leukocyte cell-cell adhesion, microglial cell activation, endothelial cell differentiation, and integrin-mediated signaling pathways.

### Unsupervised clustering for sialyation-related DEGs

**FIGURE 2:**
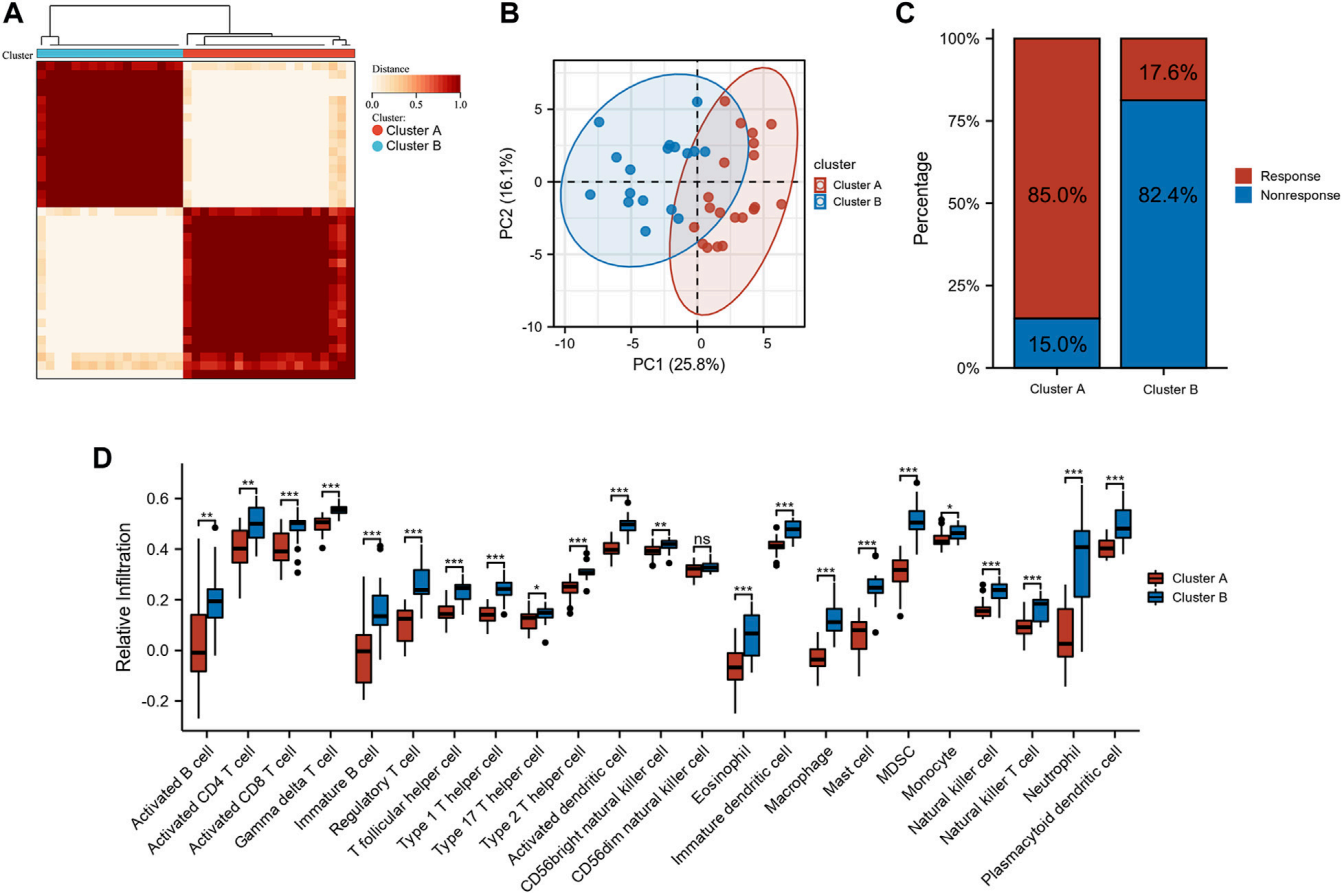
Identification of subtypes in CD. **(A)** Consensus clustering matrices of sialylation-related DEGs (k = 2). **(B)** PCA for the expression of sialylation-related DEGs to distinguish two subtypes. **(C)** Stacked bar plot of percentage of response and non-response patients in two subtypes **(D)** Infiltration fraction of immune cells in two subtypes (*p < 0.05, **p < 0.01, ***p < 0.001; ns, not significant).

Unsupervised clustering was used based on the expression files of the 40 sialyation-related DEGs. As shown in Figure 2A and Supplementary Figure S1, the two clusters had the best clustering effectiveness in the GSE16879 cohort. The transcription patterns of the sialyation-related DEGs between the two clusters differed significantly according to PCA (Figure 2B). Furthermore, we explored the number of patients who did or did not respond to anti-TNF therapy in clusters A and B. Patients in clusters A and B responded to anti-TNF therapy in proportions of 85.0% (*n* = 20) and 17.6% (*n* = 16), respectively (Figure 2C). ssGSEA was used to investigate the differences in immune infiltration between the two clusters. Figure 2D demonstrates significant differences between the two clusters of 28 immune cells; cluster B had a comparatively higher infiltration level than cluster A. Supplementary Table S3 lists the immune cell infiltration in the GSE16879 cohort.

### Identification of potential biomarkers for predicting anti-TNF therapy response

**FIGURE 3:**
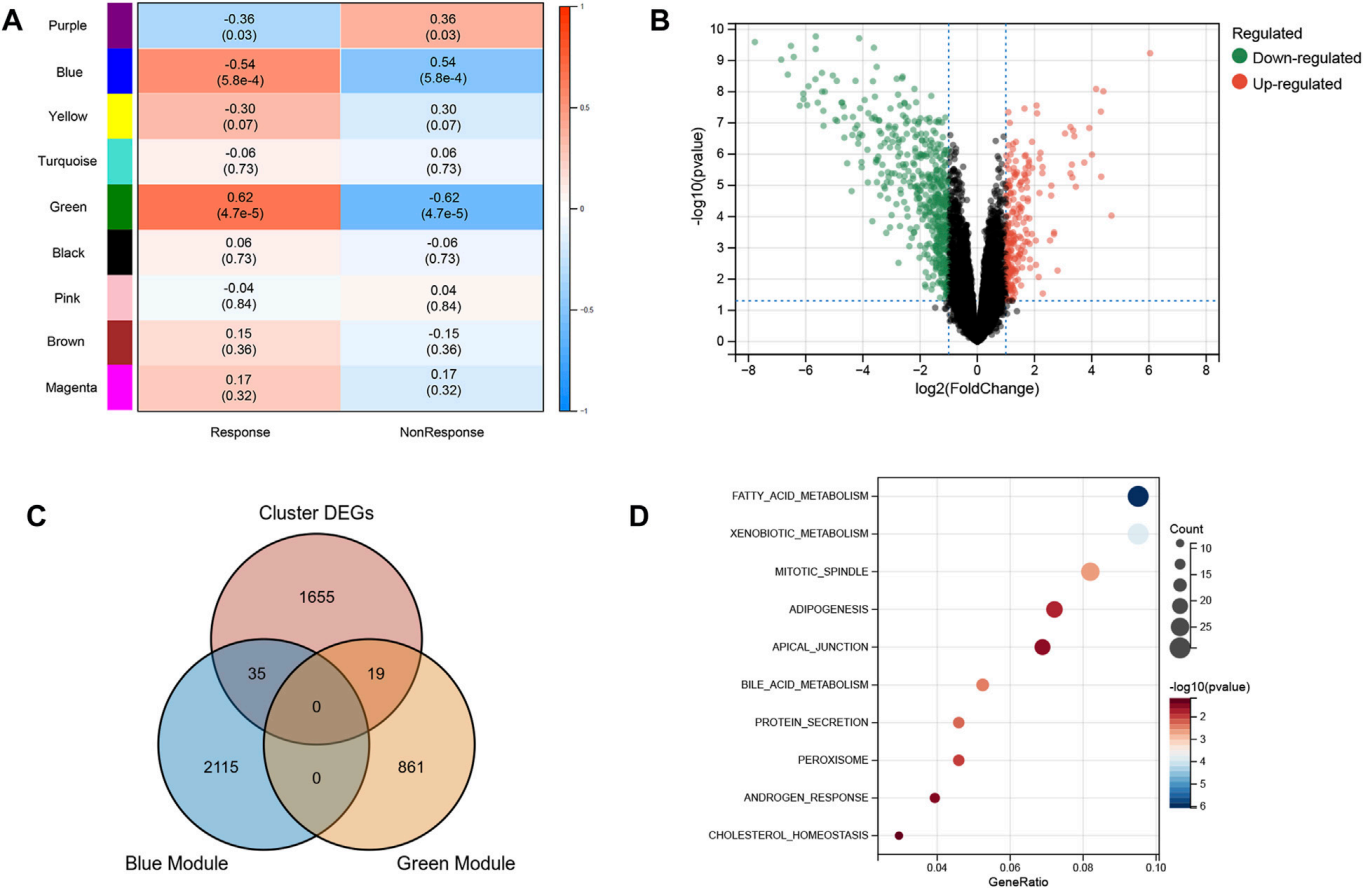
Characteristic of two subtypes. **(A)** WGCNA module trait relationships in response and non-response groups, which contained the corresponding correlation and *p*-value. Each colored row on the left represents a gene module **(B)** Volcano plot of DEGs between two subtypes. **(C)** Intersection of DEGs and response-related genes. **(D)** Biological functions of intersected genes.

Based on the excellent ability of subtypes to distinguish patients who had a response and nonresponse to infliximab, we further screened the biomarkers for predicting anti-TNF therapy response. WGCNA was used to identify modules related to anti-TNF response. To build a scale-free network, the soft threshold β was set to 2 and no scale R^2^ = 0.91. (Supplementary Figures S2A,B). Nine gene modules were identified, and the resulting gene dendrograms and module colors are presented in Supplementary Figure S2C. Figure 3A demonstrates that the blue module was negatively correlated with anti-TNF therapy response (module trait correlation = −0.54), whereas the green module had a positive correlation with anti-TNF therapy response (module trait correlation = 0.62). As patients in clusters A and B had different responses to anti-TNF treatment, the DEGs between clusters A and B were identified to explore the heterogeneity and characteristics of the two clusters (Figure 3B). Furthermore, to screen the genes related to anti-TNF treatment response, the genes in the blue and green modules were overlapped with DEGs between clusters A and B. As shown in Figure 3C, 35 and 19 overlapped genes were obtained, respectively. A total of 54 genes for further analyses. The biological functions of 54 intersecting genes were significantly enriched in metabolism-related pathways (Figure 3D).

**FIGURE 4:**
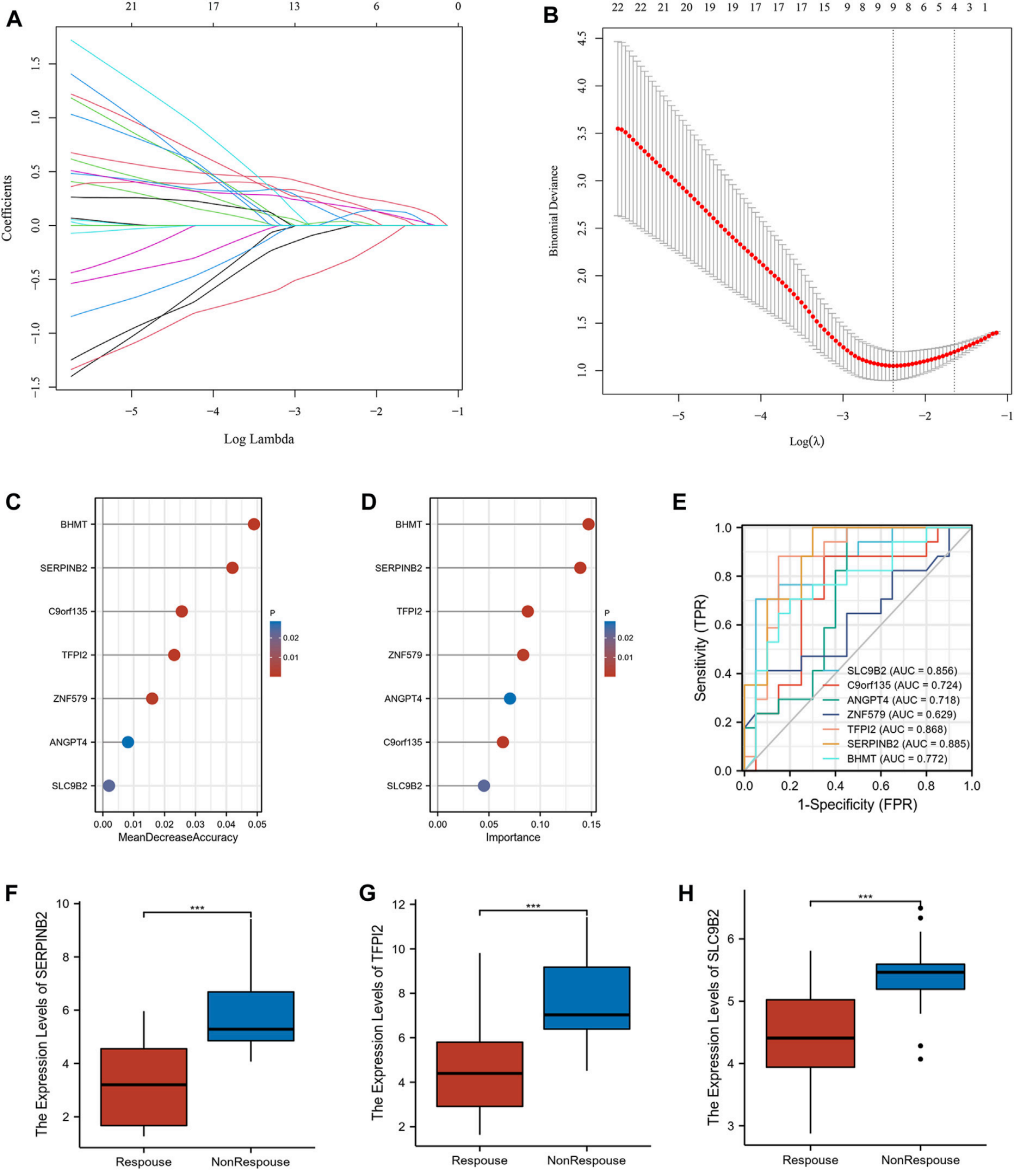
Screening the potential biomarkers. **(A)** LASSO coefficient profiles of the intersected genes. **(B)** Selection of the optimal tuning parameter (λ). **(C)** Lollipop chart of biomarkers selected by RF. **(D)** Lollipop chart of biomarkers selected by SVM-RFE. **(E)** Receiver operating characteristic (ROC) of the selected biomarkers **(F–H)** The expression levels of SERPINB2, TFPI2, and SLC9B2 in response and nonresponse groups in GSE16879, respectively. (***p < 0.001).

54 intersecting genes were used to screen he potential biomarkers using machine learning. Twenty-three genes with clinical manifestations were selected using LASSO regression (Figures 4A,B). RF and SVM-RFE were used to identify genes related to the anti-TNF response. As shown in Figures 4C,D, seven genes were selected using both the RF and SVM-RFE algorithms. We analyzed the predictive abilities of 7 genes, SERPINB2 (area under the curve, AUC = 0.885), TFPI2 (AUC = 0.868), and SLC9B2 (AUC = 0.856), which could easily distinguish responders and non-responders before treatment (Figure 4E). As shown in Figures 4F–H, the expression levels of SERPINB2, TFPI2, and SLC9B2 are significantly downregulated in response group. Therefore, SERPINB2, TFPI2, and SLC9B2 were selected for further analyses.

### Diagnostic value of SERPINB2, TFPI2, and SLC9B2 in CD

**FIGURE 5:**
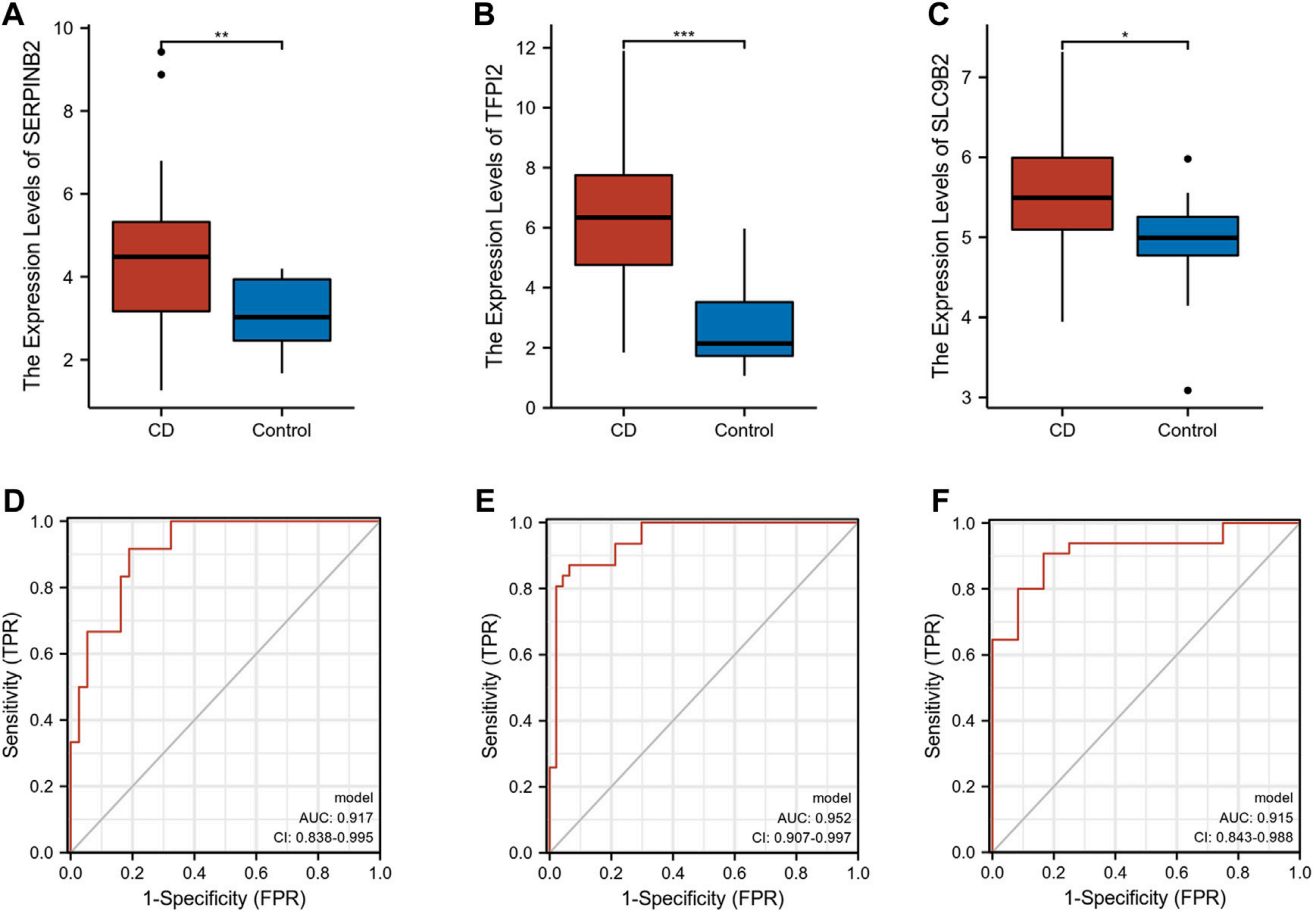
Exploration and validation of diagnostic values of selected biomarkers. **(A–C)** The expression levels of SERPINB2, TFPI2, and SLC9B2 in CD and normal tissues in GSE16879, respectively. **(D–F)** ROC of combined model in GSE16879, GSE179285, and GSE102133, respectively. (*p < 0.05, **p < 0.01, ***p < 0.001).

The expression levels of SERPINB2, TFPI2, and SLC9B2 were significantly upregulated in CD (Figures 5A–C). We further constructed a combined model using logistic regression to explore the diagnostic value of SERPINB2, TFPI2, and SLC9B2. With an area under the curve (AUC) of 0.917, the ROC of the combined model demonstrated excellent discrimination for CD diagnosis (Figure 5D). In addition, the GSE179285 and GSE102133 cohorts were used to validate the diagnostic ability of the combined model. As shown in Figures 5E,F, the AUCs of the combined model in the GSE179285 and GSE94648 cohorts were 0.952 and 0.915, respectively.

### Predictive value for Anti-TNF therapy response

**FIGURE 6:**
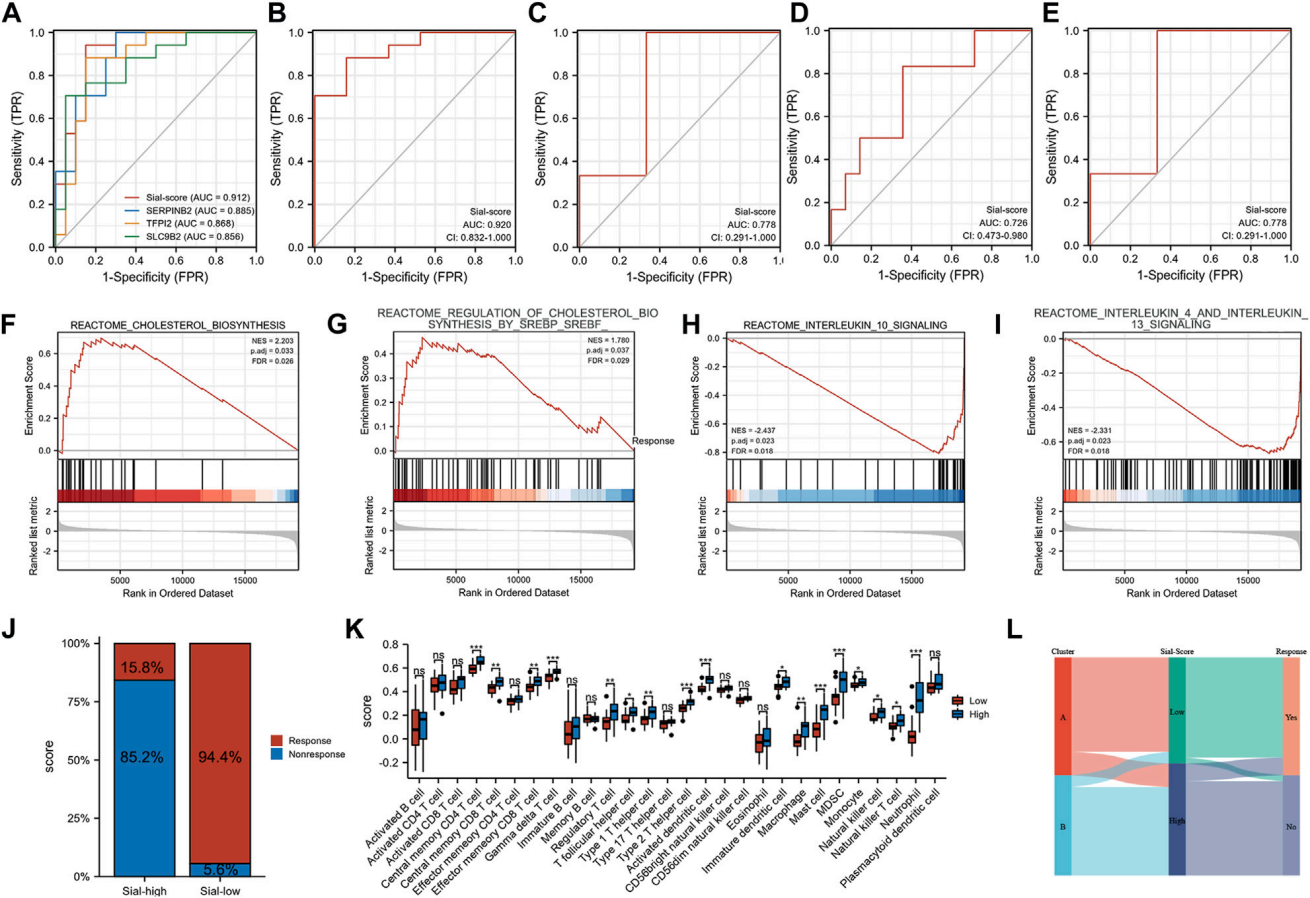
Construction of the Sial-score. **(A)** ROC of the Sial-score and selected biomarkers to distinguish responders and non-responders before treatment in GSE16879 cohort. **(B)** ROC of the Sial-score to distinguish responders and non-responders after treatment in GSE16879 cohort. **(C–E)** ROC of the Sial-score to distinguish responders and non-responders in validation cohorts GSE111761, GSE42296, and GSE107865, respectively. **(F–I)** GSEA enrichment in Sial-low group. **(F,G)** GSEA enrichment in Sial-high group. **(J)** Stacked bar plot of percentage of response and non-response patients in Sial-high and Sial-low groups. **(K)** Infiltration fraction of immune cells in Sial-high and Sial-low groups **(L)** Alluvial diagram of subtype distributions in groups with different Sial-scores and outcomes of anti-TNF therapy response. (*p < 0.05, **p < 0.01, ***p < 0.001; ns, not significant).

Furthermore, a Sial-score based on the expression levels of SERPINB2, TFPI2, and SLC9B2 was constructed to predict anti-TNF responses. As shown in Figures 6A,B, the Sial-score had outstanding discrimination for responders and non-responders to anti-TNF therapy before (AUC = 0.912) or after (AUC = 0.920) treatment in the GSE16879 cohort. Subsequently, we collected three GEO cohorts to validate the predicted values of the Sial-score. As shown in Figure 6C, the Sial-score could easily distinguish responders from non-responders to anti-TNF therapy. Interestingly, the Sial-score could predict anti-TNF response in peripheral blood samples from patients with CD in the GSE422696 and GSE107865 cohorts (Figures 6D,E).

Patients with CD in GSE16879 were divided into Sial-high and Sial-low groups based on the Sial-score. To explore the characteristics of the Sial-high and Sial-low groups, we performed GSEA using the REACTOME pathway database. As shown in Figures 6F–I, the Sial-low group was mainly associated with cholesterol biosynthesis. In contrast, the Sial-high group showed conspicuous enrichment in immune-related pathways such as interleukin 10, interleukin 4, and interleukin 13 signalings. As shown in Figure 6J, patients in the Sial-high and the Sial-low groups responded to anti-TNF therapy in 15.8% (*n* = 19) and 94.4% (*n* = 18) of the patients, respectively. Further analysis of the two groups with immune cell infiltration showed that the Sial-high group was enriched in immune cell infiltration (Figure 6K). Figure 6L illustrates the distribution of patients in the two subtypes, two Sial-score groups, and the response status to anti-TNF therapy.

## Discussion

Anti-TNF treatment helps patients with CD have better clinical outcomes, mucosal healing rates, and quality of life, although 10%–40% of individuals predominantly have no response (Cui et al., 2021). Pathophysiological heterogeneity has been a key factor limiting the outcome of new drug trials in patients with IBD over the past two decades (Bilsborough et al., 2016). However, identifying novel biomarkers is urgently needed to explore heterogeneity and provide personalized treatments for patients with CD.

Recently, Yao et al. (2022) reported that intestinal mucus sialylation by ST6GALNAC1 is critical for commensalism and bacterial metabolite homeostasis and that treatment with sialylated mucins reduces intestinal inflammation. Therefore, analyzing the expression patterns and functions of sialylation-related genes may benefit CD management. We collected data on sialylation-related genes and identified 40 genes that were differentially expressed in CD. To further explore the heterogeneity of CD, the two subtypes were analyzed using the unsupervised clustering method based on the expression of 40 sialylation-related genes. Anti-TNF therapy responders and non-responders can be distinguished by two subtypes, with subtypes with more non-responders exhibiting higher levels of immune infiltration.

To screen biomarkers for predicting the outcome of anti-TNF medication, we identified two modules related to the anti-TNF response using WGCNA. Considering the unique characteristics of the two subtypes, DEGs between the two clusters were identified and overlapped with the two modules. Furthermore, SERPINB2, TFPI2, and SLC9B2 were selected as biomarkers of anti-TNF response using LASSO, RF, and SVM-RFE algorithms. Our results also indicated that the diagnostic model combining the expression of SERPINB2, TFPI2, and SLC9B2 showed excellent performance in both the training and validation cohorts.

SERPINB2, also known as plasminogen activator inhibitor 2, is highly expressed in peripheral blood from CD patients (Burczynski et al., 2006). Wei et al. (2015) reported upregulated SERPINB2 might serve as a target gene of downregulated miR-205 to activate inflammatory signal pathways in CD rat model. In several types of cancer, including colorectal cancer, TFPI2 has been identified as a tumor suppressor gene (Li et al., 2019b). TFPI2 promoter robustly hypermethylated in the patients with CD (Kim et al., 2020). The methylation rates of TFPI2 elevated with progression of disease in inflamed colon tissue from patients with IBD, it seem to be a potential risk marker for colitis-associated cancer (Gerecke et al., 2015). SLC9B2 belongs to SLC9 family, mainly act as Na^+^/H^+^ exchangers and present in epithelial cells of the small intestine (Fuster et al., 2008; Cao et al., 2019). SLC9B2 deficiency may have an indirect effect on insulin secretion by interfering with clathrin-mediated endocytosis in β-cells (Fuster and Alexander, 2014).

To improve the clinical significance of our study, we constructed a Sial-score system based on SERPINB2, TFPI2, and SLC9B2 expression. The Sial-score has an outstanding ability to predict and classify the response to anti-TNF therapy in patients with CD before or after treatment. Importantly, only 15.8% of patients responded to anti-TNF therapy in the Sial-high group. However, 94.4% of patients with a low Sial-score responded to anti-TNF therapy. Moreover, the Sial-high group was significantly enriched in immune-related pathways and showed a high level of immune cell infiltration. This indicated that immune cell infiltration plays a role in anti-TNF resistance. Martin et al. created a cell module named GIMATS, composed of IgG plasma cells, inflammatory mononuclear phagocytes, activated T cells, and stromal cells. Cell module scores differed between non-responsive and responsive patients with CD. Monocyte-derived macrophages dominated lesions enriched in the GIMATS module (Martin et al., 2019). The failure of patients with CD to respond to the anti-TNF medication has been attributed to innate transcriptional dysregulation of monocytes, resulting in increased activation of pro-inflammatory pathways (Gaiani et al., 2020). Our results indicate that SERPINB2, TFPI2, and SLC9B2 play important roles in pathogenesis and resistance to anti-TNF therapy in CD.

However, our study has some limitations. First, these datasets lacked data regarding important clinical variables such as disease activity and duration, previous bowel resection, and smoking. Second, we used retrospective data from public databases for our research. Future prospective studies are needed to confirm our findings.

## Conclusion

In summary, a valid diagnostic model and scoring system for predicting anti-TNF therapy response was constructed based on the expression levels of SERPINB2, TFPI2, and SLC9B2. Our findings may aid auxiliary diagnoses and provide personalized treatment strategies for patients with CD.

## Data Availability

The original contributions presented in the study are included in the article/Supplementary Material, further inquiries can be directed to the corresponding authors.
