# Supplementary material for: Landscape of sialylation patterns identify biomarkers for diagnosis and prediction of response to anti-TNF therapy in crohn’s disease
Source: Front Genet. 2022 Nov 14;13:1065297. doi: 10.3389/fgene.2022.1065297 (PMC9702336; doi:10.3389/fgene.2022.1065297)
Supplement: Supplementary file 2 [file DataSheet1.docx]

Supplementary Material


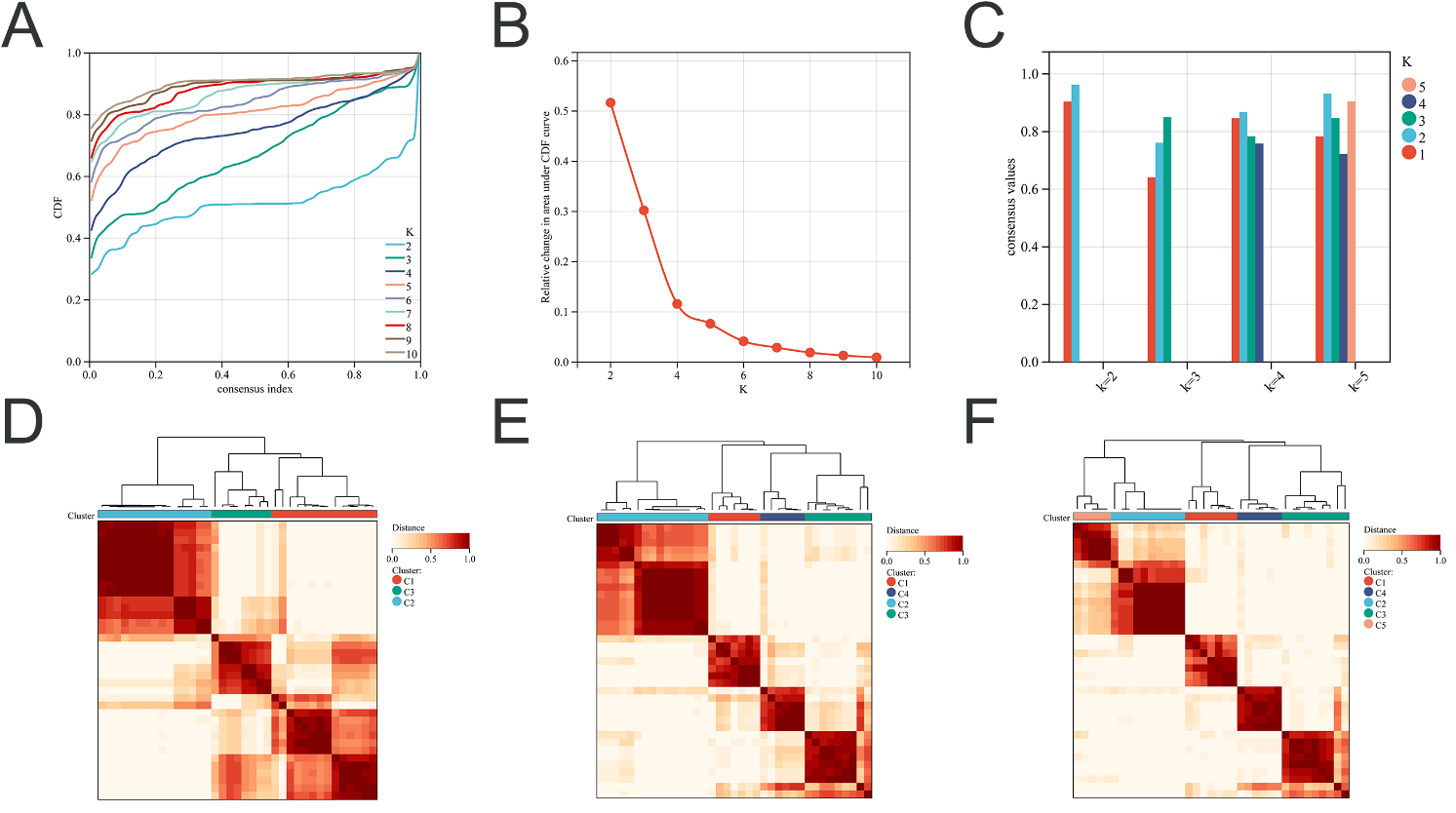


**Supplementary Figure S1.** (A) Cumulative distribution function (CDF) curves. (B) The area under CDF curves. (C) The bar plots represent the consensus scores for subtypes with k=2-5. (D-F) Consensus clustering matrices in CD merged datasets (k=3-5).


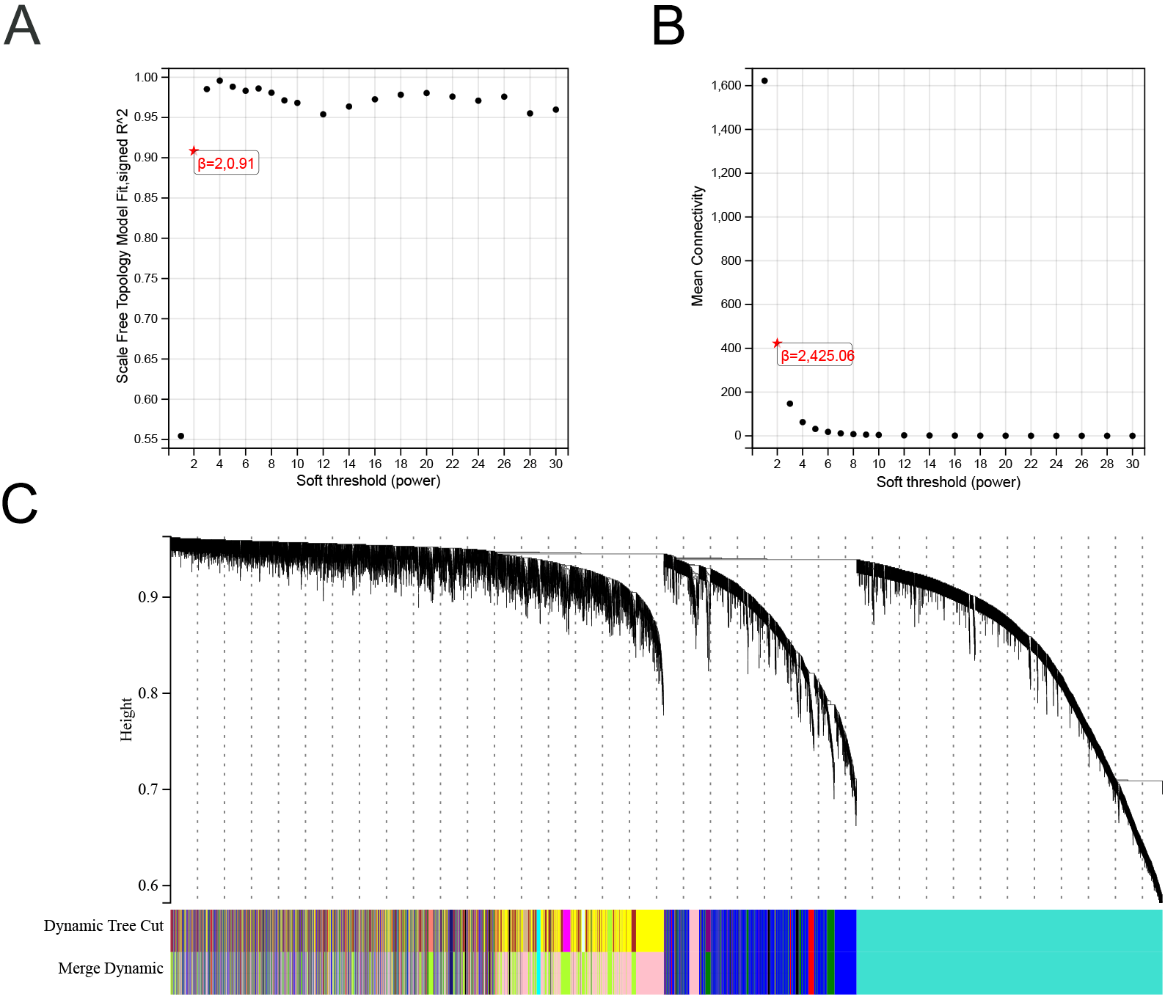


**Supplementary Figure S2.** (A-B)WGCNA soft threshold β setting. (C) Resulting gene dendrograms.
